# Borderline Resectable Pancreatic Cancer: Neoadjuvant Chemotherapy, Stereotactic Body Radiotherapy (SBRT), and Surgery

**DOI:** 10.7759/cureus.83706

**Published:** 2025-05-08

**Authors:** Abubakar Gapizov, Hadequa Noor Khan, Muhammad Subhan, Ruqiya Bibi, Ahsan Aqeel

**Affiliations:** 1 Internal Medicine, Weill Cornell Medicine, New York Presbyterian Brooklyn Methodist Hospital, Brooklyn, USA; 2 Medicine, Karachi Medical and Dental College, Karachi, PAK; 3 Medicine, Jinnah Hospital, Lahore, PAK; 4 Medicine, Allama Iqbal Medical College, Lahore, PAK; 5 Geriatrics, East Lancashire Hospitals NHS Trust, Blackburn, GBR

**Keywords:** cbd adenocarcinoma, cholangiocarcinoma, endoscopic retrograde cholangio-pancreatography, endoscopic ultrasound (eus), mrcp, obstructive jaundice, painless obstructive jaundice, plastic stent, weight loss, whipple's procedure

## Abstract

Pancreatic adenocarcinoma remains a highly fatal cancer, especially when classified as borderline resectable, characterized by limited involvement of surrounding major blood vessels that challenges but does not absolutely preclude curative surgical resection. We report the case of a 65-year-old male with a history of smoking who presented with progressive, painless jaundice, pruritus, and weight loss over 15 months. Imaging revealed intrahepatic biliary dilation, a distal common bile duct stricture, and a mass in the pancreatic head with peripancreatic lymphadenopathy. Endoscopic intervention confirmed malignant features, and stenting was performed. Subsequent cross-sectional imaging demonstrated a 3.5 cm pancreatic head mass with a 180-degree encasement of the superior mesenteric artery and aortic abutment. Carcinoembryonic antigen 19-9 (CA 19-9) was markedly elevated at 12,000 U/mL. Endoscopic ultrasound-guided biopsy confirmed pancreatic ductal adenocarcinoma. Immunohistochemical staining was positive for cytokeratin 7 and negative for CK20, supporting the pancreatic origin. The patient received six cycles of neoadjuvant-modified folinic acid (leucovorin), fluorouracil, irinotecan, and oxaliplatin (FOLFIRINOX), resulting in radiographic tumor shrinkage to 2.4 cm and a biochemical response. Stereotactic body radiation therapy (35 Gy in five fractions) focused on the vascular margin. Surgical resection via pylorus-preserving pancreaticoduodenectomy achieved R0 margins without metastasis. Postoperative recovery was uncomplicated, and the final pathology showed ypT2N1 disease with a 60% treatment response. Adjuvant chemotherapy with gemcitabine and capecitabine was initiated, and tumor markers normalized on follow-up. This case highlights the importance of a multidisciplinary, staged treatment approach in managing borderline resectable pancreatic cancer to optimize resectability and long-term outcomes.

## Introduction

Pancreatic ductal adenocarcinoma (PDAC) involving the head and uncinate process is a clinically aggressive malignancy that frequently presents with signs of biliary obstruction, such as painless jaundice and cholestatic liver enzyme elevation [1,2]. Due to the subtle onset of symptoms and the anatomical complexity of the periampullary region, diagnosis is often delayed [3]. Differentiating PDAC from other etiologies, such as cholangiocarcinoma, can be challenging in patients with distal biliary strictures, particularly when imaging and cytology findings overlap [4].

Advancements in diagnostic modalities, such as contrast-enhanced computed tomography (CT), magnetic resonance cholangiopancreatography (MRCP), and endoscopic ultrasound with fine-needle aspiration (EUS-FNA), have improved staging accuracy and treatment planning [3-5]. Endoscopic retrograde cholangiopancreatography (ERCP) remains essential for biliary decompression in patients with obstructive symptoms [6-8]. For borderline resectable PDAC, neoadjuvant chemotherapy is the standard of care to enhance resectability and improve margin status, with adjunctive radiation therapy considered in select cases [8-10].

This case highlights the multidisciplinary management of a 65-year-old male who presented with painless jaundice and was ultimately diagnosed with borderline resectable PDAC involving vascular structures. His treatment course included endoscopic biliary decompression, neoadjuvant chemotherapy, stereotactic body radiotherapy (STBR), and curative-intent pancreaticoduodenectomy. The case highlights the diagnostic intricacies and therapeutic difficulties of locally advanced pancreatic cancer.

## Case presentation

A 65-year-old male with a significant 20-pack-year smoking history presented to our tertiary care center with a 15-month history of progressive, painless jaundice accompanied by severe pruritus and significant weight loss in June 2023. The patient denied any constitutional symptoms such as fever or night sweats. However, careful history revealed the recent onset of postprandial bloating and mild steatorrhea suggestive of pancreatic exocrine insufficiency. He also denied any history of significant alcohol consumption. His past medical history was significant only for well-controlled hypertension, with no history of diabetes mellitus or chronic pancreatitis. Germline testing was not accessible, and there was no family history of pancreatic or other cancers. Table 1 shows the baseline investigations at his initial clinical presentation.

**Table 1:** Baseline laboratory investigations at initial presentation

Test	Result	Reference Range	Interpretation
Total Bilirubin	8.2 mg/dL	0.2–1.2 mg/dL	Markedly elevated (obstructive jaundice)
Direct (Conjugated) Bilirubin	6.5 mg/dL	0.1–0.3 mg/dL	Significantly elevated
Alkaline Phosphatase (ALP)	620 U/L	44–147 U/L	Elevated (cholestatic pattern)
Alanine Aminotransferase (ALT)	190 U/L	7–56 U/L	Elevated (biliary obstruction)
Aspartate Aminotransferase (AST)	170 U/L	10–40 U/L	Elevated
Gamma-Glutamyl Transferase (GGT)	710 U/L	9–48 U/L	Elevated
Carbohydrate Antigen 19-9 (CA 19-9)	12,000 U/mL	< 37 U/mL	Highly elevated (suggestive of malignancy)
Carcinoembryonic Antigen (CEA)	3.0 ng/mL	< 5.0 ng/mL	Within normal limits
Hemoglobin	11.8 g/dL	13.5–17.5 g/dL	Mild anemia
White Blood Cell Count (WBC)	9.2 ×10⁹/L	4.0 – 11.0 ×10⁹/L	Normal
Platelet Count	330 ×10⁹/L	150 – 450 ×10⁹/L	Normal
International Normalized Ratio (INR)	1.1	0.8–1.2	Normal

**Figure 1:**
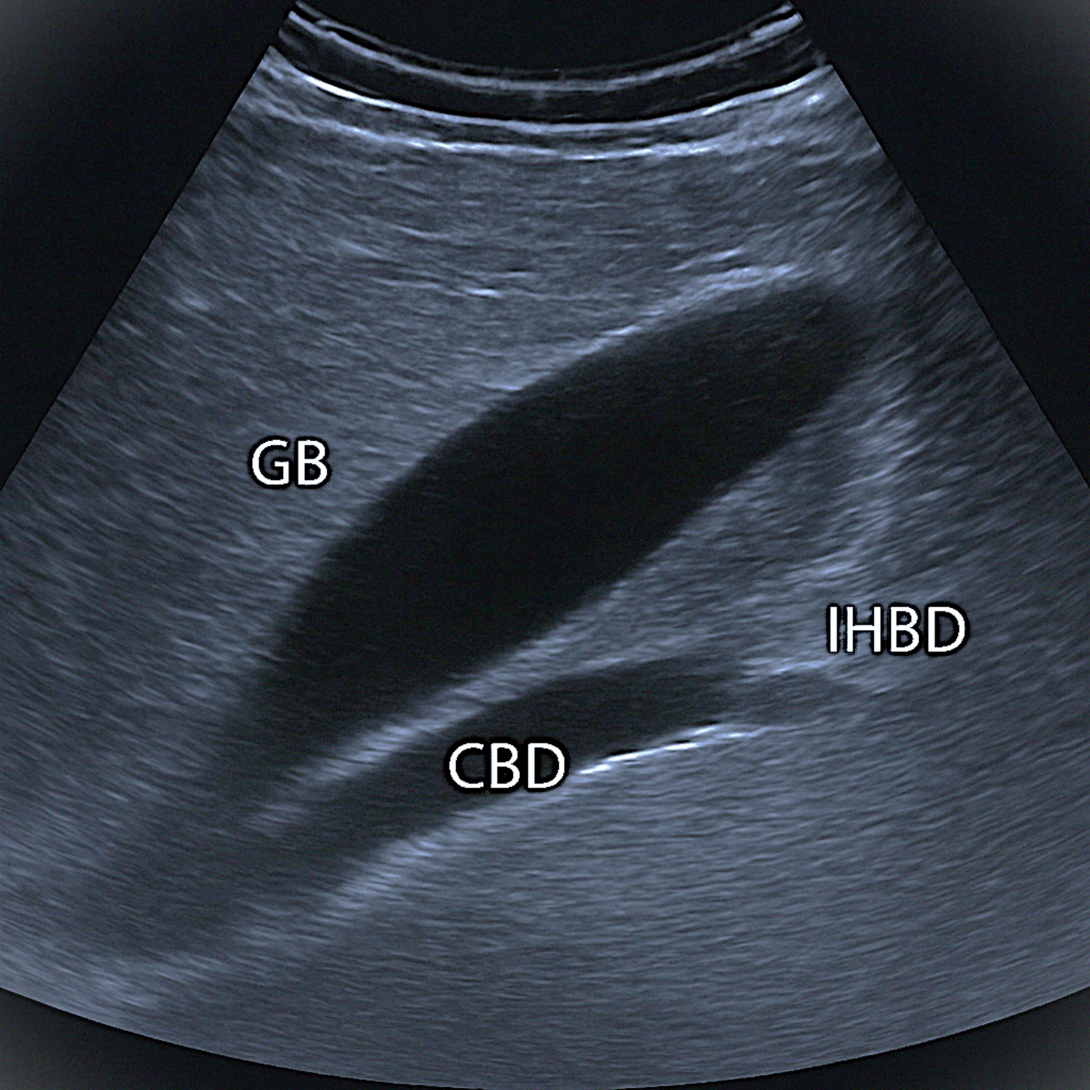
Ultrasound abdomen showing distended gallbladder and intrahepatic biliary dilatation GB: gallbladder; CBD: common bile duct; IHBD: intrahepatic biliary dilatation

As seen in Figure 1, the diagnostic workup started with an abdominal ultrasound that revealed modest intrahepatic biliary ductal dilatation with gallbladder distension.

**Figure 2:**
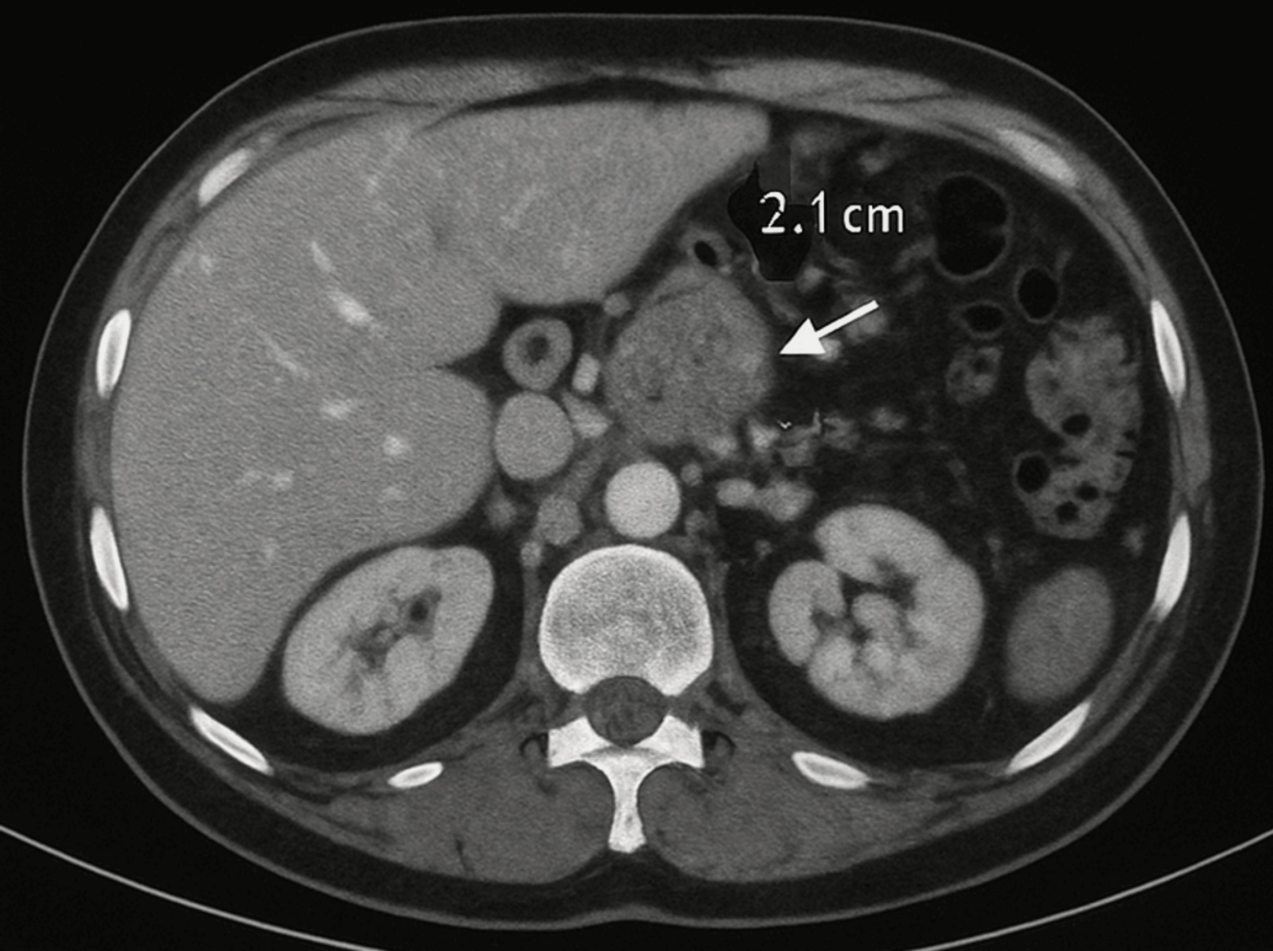
CT abdomen revealing soft tissue swelling in the head of the pancreas with peripancreatic lymphadenopathy The white arrow indicates the soft tissue swelling with its size of 2.1 cm. CT: computerized tomography

Subsequent MRCP (July 2023) revealed a 1.8 cm narrowing (stricture) in the distal common bile duct (CBD) with upstream biliary dilation (standard hepatic duct diameter 12 mm) and a markedly distended gallbladder (transverse diameter 5.2 cm). ERCP with stent placement (August 2023) confirmed a tight, irregular distal CBD stricture suspicious for malignancy, prompting placement of dual plastic stents that relieved jaundice from 8.2 to 5.6. Figure 2 shows the CT imaging obtained in October 2023; progressive biliary dilation was noted despite stenting, and new soft tissue thickening was seen in the pancreatic head (2.1 cm), enhancing the arterial phase and suspicious peripancreatic lymphadenopathy.

**Figure 3:**
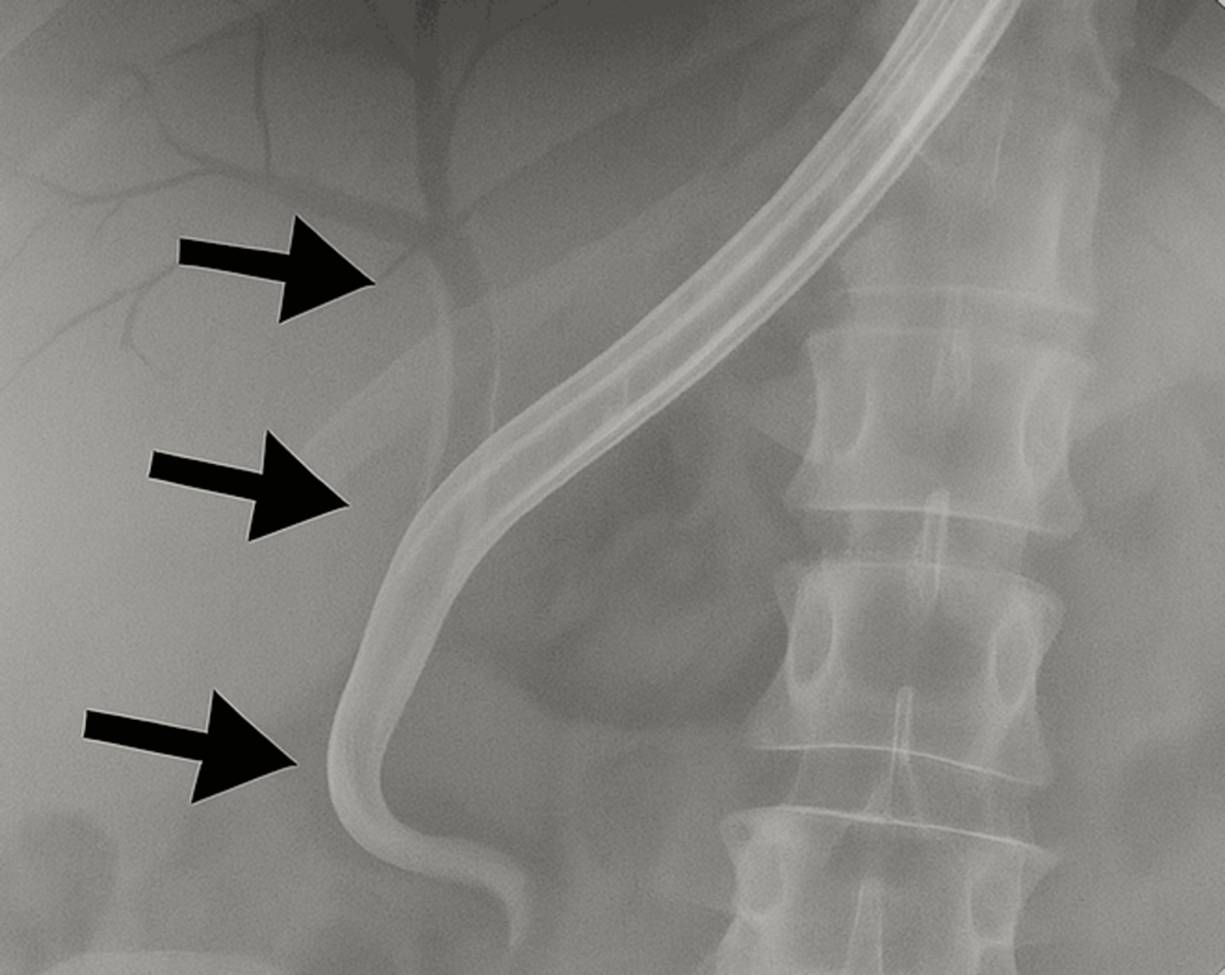
Cholangiogram showing the placement of a metallic stent in the biliary tract The black arrows show the placement of a stent in the biliary tract with its dilation up the ampulla.

Despite prior ERCP with stent placement, the patient continued to exhibit features of obstructive jaundice. In April 2024, recurrent biliary obstruction necessitated the implantation of a metallic stent. Although there was a partial decline in serum bilirubin levels following the metallic stent placement, purulent drainage from the ampulla was observed, raising concerns for superinfection or tumor necrosis complicating the clinical course. Figure 3 depicts the cholangiogram showing the placement of the metallic stent in the biliary tracts.

**Figure 4:**
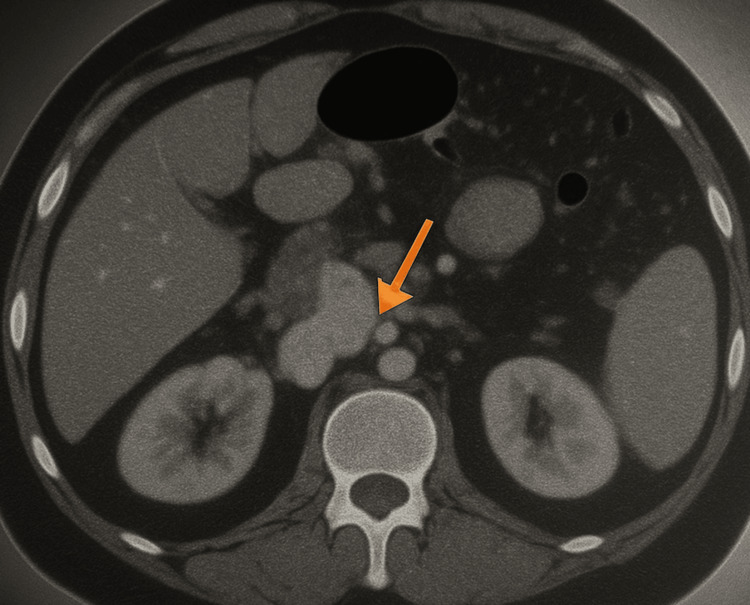
Subsequent CT abdomen showing a heterogeneous mass in the pancreas involving SMA and abutment of the aorta CT: computerized tomography; SMA: superior mesenteric artery The orange arrow indicates the pancreatic mass.

By May 2024, CT imaging demonstrated disease progression with a 3.5 cm heterogeneous mass in the pancreatic head and uncinate process, demonstrating 180° encasement of the superior mesenteric artery (SMA) and abutment of the aorta, as shown in Figure 4, radiographic features consistent with locally advanced, borderline resectable pancreatic adenocarcinoma.

**Figure 5:**
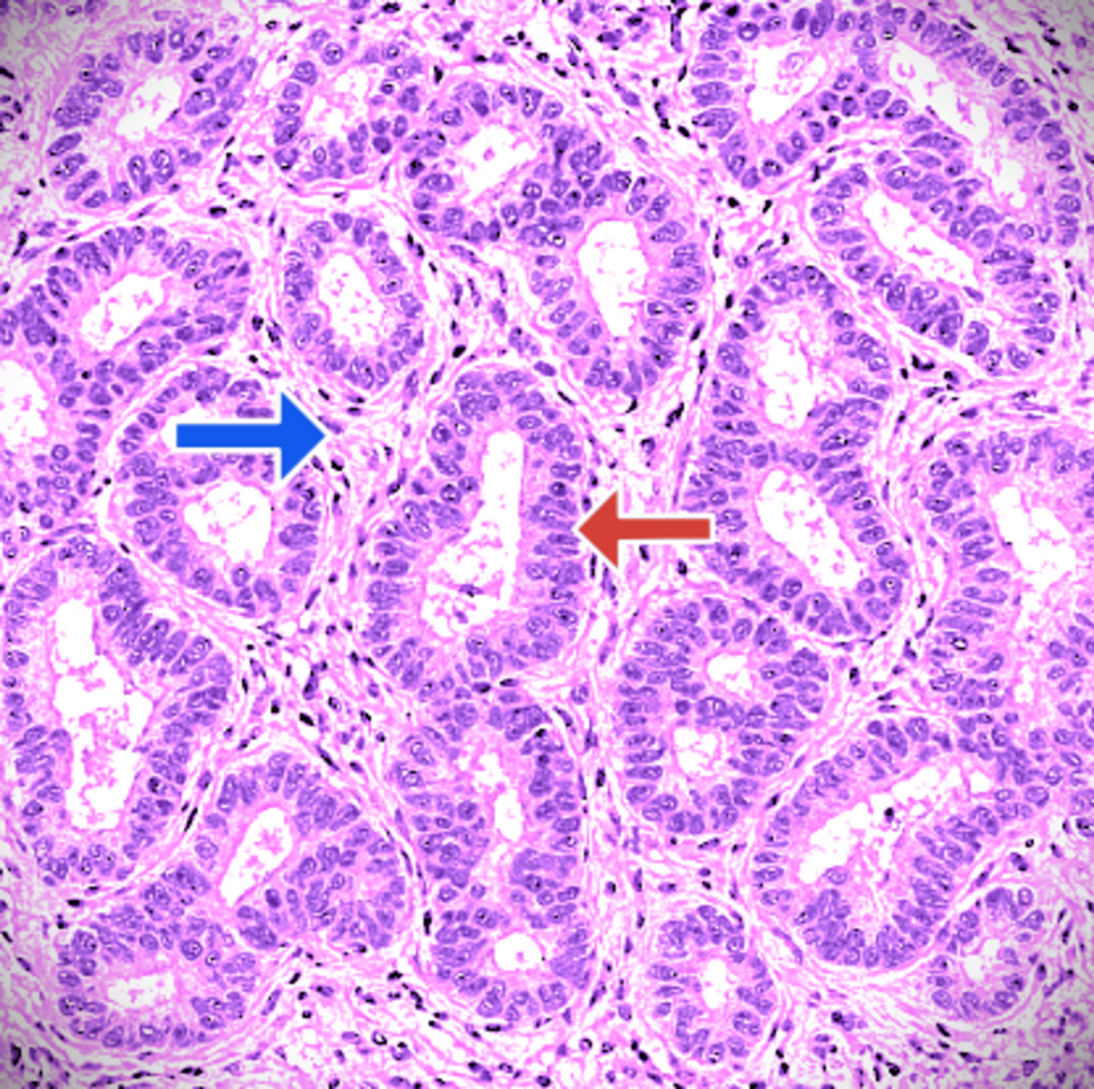
Pancreatic adenocarcinoma, EUS-FNA histology (H&E stain) EUS-FNA: endoscopic ultrasound-guided fine needle aspiration; H&E: hematoxylin and eosin The red arrow shows the malignant glandular structure; the blue arrow shows the desmoplastic stroma around these glands.

While CEA stayed normal at 2.1 ng/mL, the serum carcinoembryonic antigen 19-9 (CA 19-9) levels had sharply increased to 12,000 U/mL. Well-differentiated adenocarcinoma was confirmed by EUS-FNA, and molecular profiling was not done due to affordability and accessibility issues. Figure 5 shows the histopathology of a pancreatic mass biopsy obtained via EUS-FNA, revealing features consistent with PDAC, stained with hematoxylin and eosin. Immunohistochemical staining was positive for cytokeratin 7 (CK7) and negative for CK20, supporting the pancreatic origin.

Following a multidisciplinary tumor board review, the patient was started on neoadjuvant-modified folinic acid (leucovorin), fluorouracil, irinotecan, and oxaliplatin (FOLFIRINOX) chemotherapy (oxaliplatin 85 mg/m², irinotecan 150 mg/m², leucovorin 400 mg/m², and 5-FU 2400 mg/m² over 46 hours) with prophylactic growth factor support. After six cycles, restaging showed a partial response with a decreased tumor size to 2.4 cm and a CA 19-9 decline to 800 U/mL. He subsequently underwent SBRT (35 Gy in five fractions) targeting the SMA margin.

In October 2024, the patient underwent a successful pylorus-preserving pancreaticoduodenectomy (PPPD). Intraoperative findings included a firm 2.5 cm pancreatic head mass without peritoneal or liver metastases. Frozen section analysis confirmed R0 margins. A grade B pancreatic fistula complicated the surgical course, which was managed with percutaneous drainage, but the patient was ultimately discharged on postoperative day nine. Final pathology revealed ypT2N1 (2/28 lymph nodes) adenocarcinoma with a 60% treatment effect and negative margins. Adjuvant therapy with gemcitabine and capecitabine was initiated, with surveillance CA 19-9 normalizing to 35 U/mL by November 2024. Table 2 shows the progressive clinical events from start to end in this patient.

**Table 2:** Timeline of clinical phases and interventions in a pancreaticobiliary cancer case MRCP: magnetic resonance cholangiopancreatography; CBD: common bile duct; ERCP: endoscopic retrograde cholangiopancreatography; CT: computed tomography; CA 19–9: carcinoembryonic antigen 19–9; FOLFIRINOX: folinic acid (leucovorin), fluorouracil, irinotecan, and oxaliplatin; SBRT: stereotactic body radiation therapy; PDAC: pancreatic ductal adenocarcinoma

Date	Clinical Event
Jun 15, 2023	Initial symptoms: Jaundice, pruritus, weight loss
Jul 30, 2023	MRCP: Common bile duct (CBD) stricture identified
Aug 10, 2023	First ERCP: Dual plastic biliary stents placed
Oct 05, 2023	CT: Progressive biliary dilation and pancreatic head mass identified
Apr 15, 2024	Recurrent obstruction: Metallic biliary stent placement
May 10, 2024	Tumor progression: CA 19-9 = 12,000 U/mL
May 20, 2024	Initiated neoadjuvant FOLFIRINOX chemotherapy
Aug 01, 2024	SBRT initiated
Oct 10, 2024	Surgery: Whipple procedure (pylorus-preserving pancreaticoduodenectomy)
Nov 15, 2024	Postoperative CA 19-9 normalized

## Discussion

PDAC involving the distal CBD and large vascular structures (e.g., SMA) often poses major diagnostic and therapeutic challenges [1,2]. We describe comprehensive treatment strategies that resulted in long-term remission in a case of borderline resectable PDAC in a 65-year-old male subject who received neoadjuvant-modified FOLFIRINOX, SBRT, and PPPD prior to achieving R0 resection and experiencing long-term remission. This result provides promising evidence for the potential of aggressive intervention in multidisciplinary teams to alter the prognosis of such complex cases.

Haeberle et al. described the holistic approach and the necessity of histological confirmation, as demonstrated in our case by means of EUS-FNA for confirmatory diagnosis, showing a concurrent diagnosis of PDAC and pancreatic metastasis from renal cell carcinoma [3,4]. Qian et al. have shown an uncommon occurrence of adenocarcinoma of the gastric ectopic pancreas, emphasizing the role of robust imaging and pathology in determining tumor etiology, reflecting our own rigorous imaging and staging workup [5]. Table 3 shows a summary of the important case studies in the existing literature relevant to our case study.

**Table 3:** Summary of a few important case studies related to pancreatic ductal adenocarcinoma FOLFIRINOX: folinic acid, fluorouracil, irinotecan, and oxaliplatin; MSI-H: microsatellite instability-high; TMB: tumor mutational burden; SMA: superior mesenteric artery; SMV: superior mesenteric vein; PDAC: pancreatic ductal adenocarcinoma; RCC: renal cell carcinoma; R0 resection: complete surgical removal of a tumor with negative microscopic margins; SBRT: stereotactic body radiation therapy; 5-FU: 5-fluorouracil

Author (Year)	Patient Demographics	Tumor Characteristics	Treatment Administered	Outcome
Han et al. (2023) [6]	A 57-year-old male with metastatic PDAC and high MSI-H/TMB	Stage IV with liver metastasis, MSI-H	FOLFIRINOX → Gem/nab-paclitaxel → Ipilimumab + nivolumab	Partial response; disease stabilization on immunotherapy
Watanabe et al. (2024) [7]	Middle-aged male, unresectable locally advanced PDAC	Tumor encasing SMA, abutting SMV	Neoadjuvant chemotherapy + carbon-ion radiotherapy → conversion surgery	R0 resection achieved; disease-free at follow-up
Nakahara et al. (2023) [8]	Elderly male with mass-forming PDAC under a pancreatic lipoma	PDAC compressed by a large lipoma, head of the pancreas	Surgical resection without neoadjuvant therapy	Tumor-free margins; no recurrence at 6-month follow-up
Calvo Durán et al. (2022) [9]	A 64-year-old female with obstructive jaundice	Carcinosarcoma of the uncinate process mimicking PDAC	Pancreaticoduodenectomy + adjuvant chemotherapy	Disease-free at 3-year follow-up
Haeberle et al. (2021) [4]	A 67-year-old male with PDAC and clear cell RCC metastasis	Synchronous pancreatic ductal adenocarcinoma and RCC mets	Resection of both tumor types with multidisciplinary planning	Good postoperative recovery; short-term survival not detailed

Our case is unique due to the functionality of borderline resectable PDAC lying at the SMA, which is often considered unresectable. This led to a significant downstaging of the tumor burden by means of coordinated care, early biliary decompression, timely chemotherapy, and focused achievement of a complete resection and favorable histopathologic response. Such outcomes are rare and underscore the potential for curative treatment in challenging anatomical locations.

Our results support early ERCP for obstructive jaundice, multidisciplinary planning according to National Comprehensive Cancer Network (NCCN) recommendations, and serial tumor marker measurements to notify disease evolution, in particular CA 19-9. Postoperative CA 19-9 normalization correlated with treatment efficacy and carries important implications for surveillance strategies. Nevertheless, limitations exist. Molecular profiling (KRAS mutation status) was not actionable in this setting, echoing a major barrier to personalized oncology that has still not fully translated to the real-world data space. In addition, the single-patient nature of the report limits generalizability. This case exemplifies gaps in the literature regarding optimal sequencing and duration of neoadjuvant therapy, as well as access to molecular diagnostics in resource-poor settings. Future studies ought to investigate predictors of neoadjuvant response and prospective trials comparing treatment approaches for borderline resectable PDAC. Structured multimodal therapy can lead to curative outcomes in borderline resectable PDAC with vascular involvement. Neoadjuvant chemotherapy, radiation, and careful surgical resection remain key in their management. Access to personalized treatments and further studies of neoadjuvant approaches is paramount to improving survival outcomes in these complex cases.

## Conclusions

This case underscores the importance of a multidisciplinary, stepwise approach in managing borderline resectable PDAC involving the distal CBD. Early biliary decompression, precise staging, and timely initiation of neoadjuvant-modified FOLFIRINOX followed by SBRT facilitated successful curative resection via PPPD. A marked postoperative decline in CA 19-9 levels highlighted the utility of serum tumor markers in monitoring therapeutic response. In resource-limited settings, coordinated care pathways and guideline-based protocols remain crucial for optimizing outcomes. Future integration of precision medicine and broader access to multidisciplinary tumor boards may further enhance surgical candidacy and survival in aggressive pancreatic malignancies.
